# Graphene Decorated with Iron Oxide Nanoparticles for Highly Sensitive Interaction with Volatile Organic Compounds †

**DOI:** 10.3390/s19040918

**Published:** 2019-02-22

**Authors:** Marius Rodner, Donatella Puglisi, Sebastian Ekeroth, Ulf Helmersson, Ivan Shtepliuk, Rositsa Yakimova, Andreas Skallberg, Kajsa Uvdal, Andreas Schütze, Jens Eriksson

**Affiliations:** 1 Applied Sensor Science Unit, IFM, Linköping University, 58183 Linköping, Sweden; donatella.puglisi@liu.se (D.P.); jens.eriksson@liu.se (J.E.); 2 Plasma & Coatings Physics Division, IFM, Linköping University, 58183 Linköping, Sweden; sebastian.ekeroth@liu.se (S.E.); ulf.helmersson@liu.se (U.H.); 3 Semiconductor Materials Division, IFM, Linköping University, 58183 Linköping, Sweden; ivan.shtepliuk@liu.se (I.S.); rositsa.yakimova@liu.se (R.Y.); 4 Division of Molecular Surface Physics & Nanoscience, IFM, Linköping University, 58183 Linköping, Sweden; andreas.skallberg@liu.se (A.S.); kajsa.uvdal@liu.se (K.U.); 5 Lab for Measurement Technology, Department of Systems Engineering, Saarland University, 66041 Saarbrücken, Germany; schuetze@lmt.uni-saarland.de

**Keywords:** epitaxial graphene, metal oxide nanoparticle, gas sensor, volatile organic compounds, benzene, formaldehyde, derivative sensor signal, air quality sensor

## Abstract

Gases, such as nitrogen dioxide, formaldehyde and benzene, are toxic even at very low concentrations. However, so far there are no low-cost sensors available with sufficiently low detection limits and desired response times, which are able to detect them in the ranges relevant for air quality control. In this work, we address both, detection of small gas amounts and fast response times, using epitaxially grown graphene decorated with iron oxide nanoparticles. This hybrid surface is used as a sensing layer to detect formaldehyde and benzene at concentrations of relevance (low parts per billion). The performance enhancement was additionally validated using density functional theory calculations to see the effect of decoration on binding energies between the gas molecules and the sensor surface. Moreover, the time constants can be drastically reduced using a derivative sensor signal readout, allowing the sensor to work at detection limits and sampling rates desired for air quality monitoring applications.

## 1. Introduction

Several toxic air pollutants in more than 80% of the urban areas where air pollution is monitored exceed the World Health Organization’s (WHO) recommended safe exposure levels. Poor air quality has been associated with several negative health aspects ranging from less severe conditions, such as skin and eye irritation, to more acute respiratory problems, cancer, or death. Air pollution has been estimated to cause 8 million annual deaths and to financially burden the European region by about 1.6 trillion US dollars per year [1,2].

Air quality (AQ) monitoring and control using extremely sensitive sensors are crucial from the viewpoint of preventing further deaths and diseases correlated with toxic air substances. However, commercial sensors/instruments available today are either large, expensive, and complex or small but limited by poor selectivity, sensitivity, and a slow sampling rate [3]. In addition, there are no commercially available sensors with sufficiently low detection limits to monitor carcinogenic volatile organic compounds (VOCs), such as formaldehyde (CH_2_O) and benzene (C_6_H_6_), at levels of relevance to human health. Benzene is a genotoxic aromatic compound, especially associated with leukemia. There are no safe exposure limits for C_6_H_6_ according to the last published WHO recommendations [4], however the European Air Quality Directive guidelines [5] recommend an exposure limit of 1.6 parts per billion (ppb), and in France, which has very strict guidelines, the limit is 0.6 ppb [6].

In the future, AQ assessment should ideally be based on real-time monitoring of air pollutants with high spatial resolution [7], allowing pollution mapping and forecasting. This can only be satisfied by utilizing low-cost monitoring devices of small size. Szulczynski et al. [3] recently published a review of currently commercially available sensors for VOC detection in outdoor and indoor air, in which they concluded that current sensor technologies suffer from too high limit of detection or poor selectivity.

In a report by Spinelle et al. from 2017, commercially available portable low-cost sensors for VOCs were reviewed, and it was found that few sensors can detect C_6_H_6_ at the concentrations of relevance for AQ monitoring [8], with most sensors showing detection limits that are at least one order of magnitude above the guideline levels. Some of these small sensors can reach a low C_6_H_6_ detection limit down to 0.5 ppb. Unfortunately, none of the sensors are selective to a particular VOC, making it impossible to distinguish, e.g., the C_6_H_6_ concentration. Some of the reviewed sensors also include a selective absorbing cartridge for benzene. Unfortunately, the best limit of detection for commercially available sensors selective to C_6_H_6_ was found to be 10 ppb for the Ion Science Tiger Select (Ion Science, Great Britain).

Among state-of-the-art research studies on portable AQ sensors [9], there are reports of prototypes including gas sensitive field effect transistors based on silicon carbide (SiC-FETs) that allow detection of 1–3 ppb of C_6_H_6_ [10]. Both SiC-FETs [11] and metal-oxide based sensors [12] operated in Temperature Cycled Operation can yield selectivity to specified VOCs. A drawback of this approach is the required sampling time, which increases the response time. An approach to improve the detection limit even further is the use of pre-concentrators [13], in which the gas concentration is temporarily increased by adsorbing gas over a long time and then releasing it. A similar approach is used by Trzcinski et al., where a miniaturized photoionization detector is coupled to a specifically designed pre-concentrator with selective desorption of benzene [14]. However, also those approaches have a prolonged response time as a drawback.

A low cost, portable sensor capable of detecting benzene at 1 ppb or lower concentrations would thus constitute a breakthrough in the field of air quality monitoring.

Using the unique properties of graphene as a transducer allows fabrication of sensor devices that can be used for gas detection where low concentrations can be detected, including air quality control for human health. Besides a high sensitivity, also interaction with specific target analytes and a good selectivity must be addressed to get a useful sensor device. It has already been shown that decoration of the graphene surface with metal/oxide nanoparticles can lead to a higher sensitivity and selectivity towards certain gases, e.g., nitrogen dioxide (NO_2_), C_6_H_6_ and CH_2_O [15,16]. Using a “soft” decoration approach, the surface chemistry of the sensing layer could be modified without changing the transducer’s electronic properties. In addition, zinc oxide (ZnO) fibers [17] and iron oxide (Fe_3_O_4_) decorated multiwall carbon nanotubes [18] in combination with ultraviolet (UV) irradiation have shown sensitivity towards C_6_H_6_. However, poor reproducibility and insufficient detection limits make the state-of-the-art sensors ill-suited for human safety applications. So far, these sensors were able to detect the mentioned gases down to tens of ppb, but not reliably lower. In this paper, which is an extension of a conference contribution [19], we demonstrate how it is possible to detect even single ppb concentrations, and we further introduce a data evaluation approach allowing fast response times to meet the criteria for AQ monitoring.

## 2. Materials and Methods

### 2.1. Sensing Layer Preparation

The graphene was grown epitaxially on silicon carbide (SiC) through a sublimation process were an on-axis, semi-insulating (0001) 4H-SiC substrate (7 mm × 7 mm) is used for the formation of graphene in argon (Ar) at a temperature of 2000 °C and a pressure of 1 bar [20]. The method allows a highly homogenous growth of monolayer graphene and requires no further transfer to another insulator. Hollow cathode pulsed plasma sputtering [21] was used to functionalize the graphene surface with iron oxide nanoparticles (NP).

### 2.2. Characterization Techniques

Before and after the deposition of nanoparticles, a series of characterization measurements was conducted to determine the graphene uniformity and quality, and to see if any damage of the graphene occurred during the deposition. Atomic Force Microscopy (AFM) (Quadrexed Dimension 3100 with a Nanoscope IVa controller) was used in tapping mode to obtain topography images of the sensing layers. The measurements were performed using silicon (Si) tips (PPP-NCHR-50 from Nanosensors) with a tip radius of curvature below 7 nm. X-ray photoelectron spectroscopy (XPS) studies using a Microlab 310-F spectrometer were performed to investigate possible alterations made to the sample after deposition of NPs and to establish if Fe_3_O_4_ was present on the surface.

### 2.3. Sensor Device Fabrication

As a first step, titanium on gold (Ti/Au, 2/200 nm) contact pads were thermally evaporated onto the epitaxially grown graphene on SiC (EG/SiC) before functionalization. The contacts have a size of 1 mm × 1 mm with a distance of 1 mm between them. The sensor chip and a Pt-100 resistance thermometer were glued (Aremco Ceramabond 571) onto a ceramic heater (Heraeus GmbH) to enable a controlled temperature loop, and welded to a 16 pin TO8 header to establish the electrical contacts. This device was mounted on top of a TO8-socket and connected to its pins using gold-wire bonding and silver glue (Epotek E3081). The final sensor was placed in a flow chamber connected to a gas mixing setup. A Keithley 2601B SourceMeter was used in a two-wire mode to measure the resistance between the contacts during gas exposure. The total gas flow was kept constant at 100 mL/min, and a dry mixture of 80% N_2_ and 20% O_2_ was used as a purging and carrier gas. A more detailed description of the measurement system can be found in a previous work [22]. 

### 2.4. Theoretical Approach

The adsorption of gas molecules (C_6_H_6_ and CH_2_O) on pristine EG (PEG) and Fe_3_O_4_-decorated epitaxial graphene (DEG) on Si-face 4H-SiC was investigated based on hybrid gas-phase density functional theory (DFT) calculations performed by using Gaussian 16 Rev. B.01 program package [23]. As a model of PEG, 4 × 5 first graphene layer located above 4 × 5 buffer layer, which is covalently bonded to 4 × 4 Si-face surface of hexagonal SiC, has been chosen. DEG was simulated by full geometrical optimization of Fe_3_O_4_ located on PEG. All dangling bonds are passivated by the hydrogen atoms. The calculations were carried out using M06-2X level of theory with consideration of split basis set [24]. It is important to note that the dispersion-corrected DFT functional M06-2X includes implicitly modified parameters associated with the Hartree-Fock exchange interaction, thereby allowing prediction of the weak van der Waals interaction [25]. A 6-31G basis set was used for carbon (C), silicon (Si), oxygen (O) and hydrogen (H) atoms, while a LANL2DZ (Los Alamos National Laboratory 2 Double-Zeta) basis set was utilized for Fe species [26]. All atoms were enabled to be fully relaxed during geometrical optimization. All calculations were carried out without symmetry restrictions. 

The adsorption energy of gas molecules (*E*_ads_) was calculated by using the following Equation:

(1) $$E_{\mathrm{ads}}=\left(E_{\mathrm{tot}}^{\mathrm{PEG}/\mathrm{DEG}}+E_{\mathrm{tot}}^{\mathrm{gas}}\right)-E_{\mathrm{tot}}^{\mathrm{gas}@\mathrm{PEG}/\mathrm{DEG}}$$

where $E_{\mathrm{tot}}^{\mathrm{PEG}/\mathrm{DEG}}$ and $E_{\mathrm{tot}}^{\mathrm{gas}}$ are the total energies of isolated templates and gas molecules (C_6_H_6_ or CH_2_O), respectively, whereas $E_{\mathrm{tot}}^{\mathrm{gas}@\mathrm{PEG}/\mathrm{DEG}}$ is the total energy of the PEG or DEG after complexation with gas molecules. Counterpoise correction for basis set superposition error (BSSE) [27] was applied for accurate prediction of the adsorption energy. 

## 3. Results and Discussions

### 3.1. Morphological and Structural Characterization

**Figure 1:**
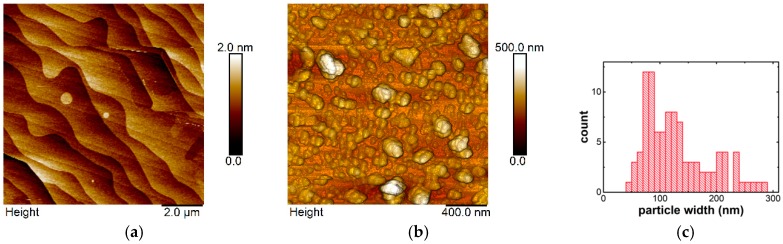
Atomic Force Microscopy (AFM) image of the graphene sensor surface before (**a**) and after (**b**) decoration with Fe_3_O_4_ NPs, and (**c**) the particle width distribution.

Figure 1 shows AFM graphs of the graphene sensor surface before (a) and after (b) decoration with Fe_3_O_4_ NPs. Neglecting the characteristic steps corresponding to the SiC step bunching (typically 0.5–1.5 nm in height), the as-grown graphene surface in Figure 1a shows almost no roughness (Rq ≈ 0.25 nm on the terraces). The particle coverage is about 60%, and single particles have an average diameter of about 80 nm. The histogram in Figure 1c shows the width distribution for single particles and particle clusters. The main peaks arise for particles between 70 and 90 nm, but also much larger agglomerates up to 290 nm can be seen. Raman spectroscopy confirms structural integrity of the graphene surface also after the decoration [16]. 

**Figure 2:**
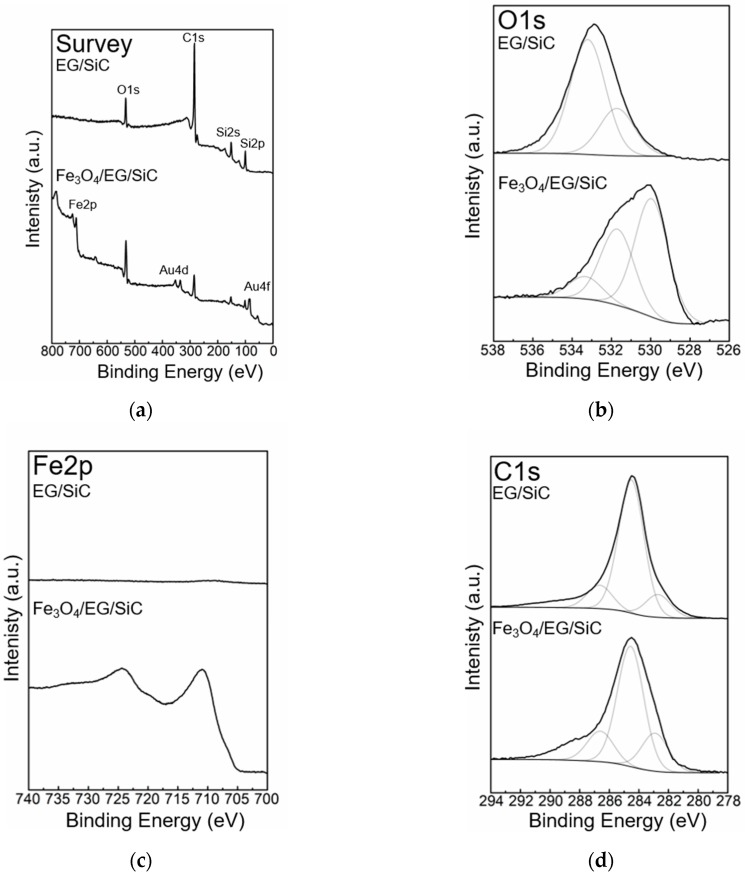
X-ray photoelectron spectroscopy (XPS) survey spectra (**a**), O1s (**b**), Fe2p (**c**) and C1s (**d**) XPS core level spectra for as-grown EG/SiC and Fe_3_O_4_ NP deposited on graphene (Fe_3_O_4_/EG/SiC).

The effect of deposition of Fe_3_O_4_ NPs onto graphene was investigated by means of XPS. The elemental compositions of as-grown EG/SiC prior to and after deposition of Fe_3_O_4_ NPs (Fe_3_O_4_/EG/SiC) were obtained to demonstrate the presence of Fe_3_O_4_ NPs on the latter sample. The XPS survey spectra for EG/SiC sample and Fe_3_O_4_/EG/SiC sample are shown in Figure 2a. The XPS survey spectrum for Fe_3_O_4_/EG/SiC showed the presence of iron and gold (Au) with XPS peaks Fe2p, Au4d, and Au4f found at binding energy position about 711 eV, 340 eV and 84 eV [28]. The gold peaks in the XPS spectra come from the Ti/Au contacts (see Section 2.3). Both samples also showed the presence of oxygen, carbon, and silicon, see Figure 2 with XPS peaks O1s, C1s, Si2s and Si2p at binding energy positions about 532 eV, 285 eV, 151 eV and 100 eV respectively. XPS O1s, Fe2p, and C1s core level spectra for EG/SiC and Fe_3_O_4_/EG/SiC are shown in Figure 2b–d. Quantification of oxygen and carbon content for EG/SiC and Fe_3_O_4_/EG/SiC, based on the XPS O1s and C1s core level spectra has been performed. An increase of O, estimated to be a fivefold increase, and a decrease of C, reduced to about half for Fe_3_O_4_/EG/SiC, were observed. The strong increase in oxygen signal is shown for Fe_3_O_4_/EG/SiC sample, with an additional peak at binding energy position 530.0 eV, in good agreement with the presence of the chemical structure Fe-O in Fe_3_O_4_ NPs. XPS Fe2p core level spectra before and after deposition of Fe_3_O_4_ NPs are shown in Figure 2c. As expected, no iron could be detected for EG/SiC. After deposition, two strong peaks at binding energy position 710.9 eV and 724.4 eV were observed for Fe_3_O_4_/EG/SiC. The corresponding XPS C1s core level spectra for EG/SiC and Fe_3_O_4_/EG/SiC are presented in Figure 2d.

### 3.2. Gas Measurements

**Figure 3:**
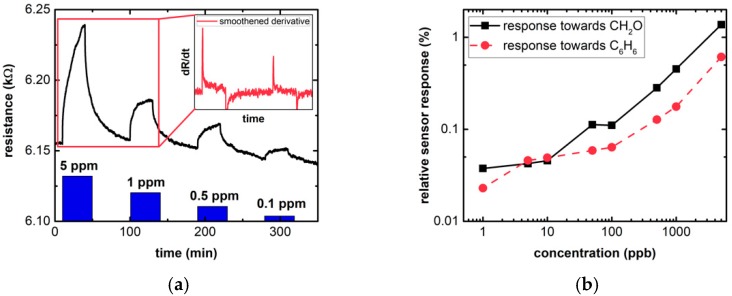
(**a**) Sensor behavior exemplarily shown for the first four exposures (5 ppm–0.1 ppm, 30 min exposure and relaxation) towards formaldehyde at 150 °C in dry air with the smoothened derivative signal as inset and (**b**) relative sensor response towards different concentrations of formaldehyde and benzene.

After the successful decoration of the graphene surface with Fe_3_O_4_ NPs, gas measurements were performed. Figure 3a shows how the resistance of the sensor changes with the exposure towards CH_2_O. The sensor was exposed to CH_2_O and C_6_H_6_ in a dry background of synthetic air using concentrations ranging from 5 parts per million (ppm) to 1 ppb, and a pulse duration of 30 min. Tests to identify a good operating temperature have been conducted and 150 °C, which is a good compromise between stability and high response, was chosen for all measurements shown here. Similar measurements with pure graphene did not show any response to both test gases. In an earlier work of ours [16], it has already been shown that Fe_3_O_4_ nanoparticle-decorated graphene sensors can be used for formaldehyde and benzene detection, but not over such a large range and down to a single ppb. The same measurement was repeated twice in the same environment and was repeated once more approximately one year later with the same sensor. The measured responses show some small deviations between the measurements without a clear trend of decreasing response over time. The sensor response shown here is from the first measurement. The response is defined as $\frac{R-R_{0}}{R_{0}}$, where *R* is the saturated resistance signal and *R*_0_ corresponds to the baseline resistance before the gas exposure. The raw sensor signal in Figure 3a shows that the sensor does relax completely, but a slow drift hinders the baseline resistance to stay stable over time. DFT calculations support the choice of operating temperature, as they show that it is possible to overcome the desorption barrier of C_6_H_6_ at the sensor surface at 150 °C (see Figure S1). The relative response for different concentrations of the two target gases is shown in Figure 3b. A distinct response for both gases over the whole range can clearly be observed. Both formaldehyde and benzene can be quantitatively measured down to a single ppb. The relative responses towards 1 ppb CH_2_O and C_6_H_6_ are about 0.04% and 0.02%, respectively. It is worth to be noticed that the WHO recommended safety limit for CH_2_O (81 ppb over 30 min of exposure) is easily reached with a relative response above 0.1%.

Besides a very good sensitivity, also time constants of these measurements need to be addressed. For an application in indoor air monitoring, the time constants for the sensor should be in the range of half a minute to several minutes, depending on the application. However, as shown in Figure 3a, even an exposure towards the gas over 30 min does not lead to a saturated sensor response. Using the time it takes for the first order time derivative of the sensor signal to reach its maximum for each exposure instead, the time constant can be decreased significantly. This is exemplarily shown for the first two exposures in the inset in Figure 3a, comparing the raw sensor signal with *dR*/*dt* (after smoothening the data with a 500-point moving average filter), where the time derivative signal reaches its maximum value after about 50 s. It is evident from the insert that *dR*/*dt* exhibits distinct peaks in response to CH_2_O and the peak values are concentration dependent. Because of the smaller time constants needed to reach maximum *dR*/*dt*, the actual sensor signal does not have to be saturated when using the rate of change instead of the absolute value of resistance. It is therefore possible to significantly improve the speed of the sensor by utilizing *dR*/*dt* as a sensor parameter. 

### 3.3. DFT Calculations

**Figure 4:**
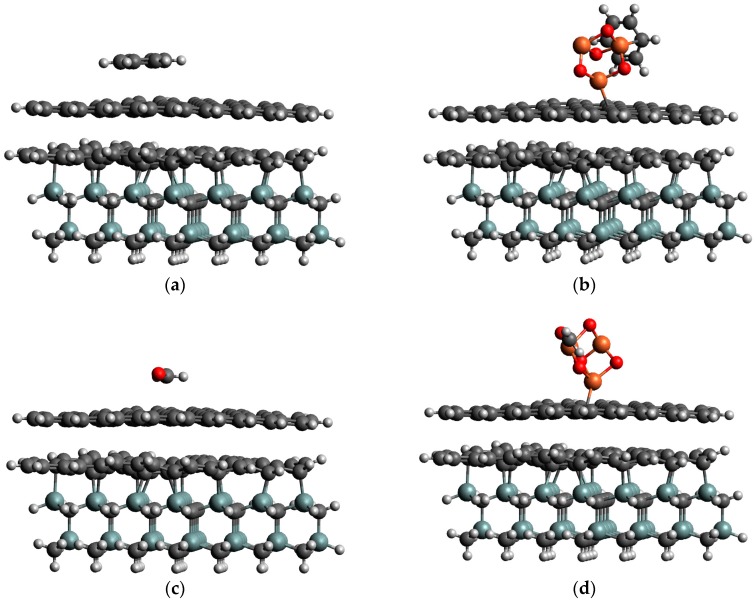
Optimized adsorption configurations of benzene on (**a**) PEG and (**b**) DEG. Preferred adsorption geometries of formaldehyde on PEG and DEG are presented as (**c**) and (**d**).

An efficiency of the decoration approach towards gas sensing can be directly demonstrated and validated by the DFT results. For this aim, a comparative analysis of the adsorption of benzene and formaldehyde onto non-decorated and Fe_3_O_4_-decorated epitaxial graphene was performed. Energetically favored adsorption configurations of considered gas molecules on PEG and DEG are illustrated in Figure 4. The adsorption energy for C_6_H_6_ on DEG, *E*_ads_ = 1.795 eV, is significantly higher than on PEG with a value of *E*_ads_ = 0.284 eV. In the absence of the Fe_3_O_4_ nanoparticle, C_6_H_6_ molecule adsorbs in the flat geometry, which is parallel to the surface. In this case, the center of C_6_H_6_ ring lays on top of a carbon atom belonging to graphene. On the other hand, a functionalization of graphene with Fe_3_O_4_ causes the formation of a strong chemical bond between C_6_H_6_ ring and one of the Fe atoms, facilitating the C_6_H_6_ adsorption in the tilted geometry regarding the graphene surface. Like the situation for C_6_H_6_, we found that CH_2_O molecule prefers to accommodate in a parallel geometry to graphene surface with weak adsorption energy of 0.149 eV, while the energetically preferred adsorption mode for CH_2_O on DEG is related to the formation of strong Fe-O covalent bond with a bond length of the 1.98 Å. The calculated adsorption energy of 1.870 eV is much higher than the value predicted for adsorption on pristine graphene sample, implying the strong chemisorption case. To summarize, one can conclude that weak physisorption of gas molecules on non-decorated graphene is immediately changed to strong chemisorption when iron oxide NPs are involved in adsorption phenomena. From an experimental point of view, the observed chemical similarity between adsorption configurations of benzene and formaldehyde enables us to anticipate comparable responses of Fe_3_O_4_-decorated epitaxial graphene to exposure of both gases, with slightly higher sensitivity towards formaldehyde.

## 4. Conclusions

Benzene and Formaldehyde sensing properties were investigated using epitaxially grown graphene on silicon carbide decorated with Fe_3_O_4_ NPs. We could verify the decoration and the integrity of the graphene surface using AFM and XPS. With this sensor platform, concentrations down to a single ppb of toxic VOCs could be quantitatively measured, which makes it very promising for air quality monitoring. DFT calculations show that the gas molecules are more likely to bind to the decorated sensor surface. Moreover, by evaluating the first-order time derivative of the sensor signal, it was possible to significantly enhance the speed of the sensor, yielding response times of less than one minute, in turn allowing sampling rates desired in air quality monitoring.

## Supplementary Materials

The following are available online at https://www.mdpi.com/1424-8220/19/4/918/s1, Figure S1: Reaction energy profile corresponding to benzene desorption. IS, TS and FS represent the initial state, transition state and final state, respectively.
