# Supplementary material for: Graphene Decorated with Iron Oxide Nanoparticles for Highly Sensitive Interaction with Volatile Organic Compounds
Source: Sensors (Basel). 2019 Feb 22;19(4):918. doi: 10.3390/s19040918 (PMC6413193; doi:10.3390/s19040918)
Supplement: Supplementary file 1 [file sensors-19-00918-s001.pdf]

<u>Supplementary information</u>

Graphene Decorated with Iron Oxide Nanoparticles for Highly Sensitive Interaction with Volatile Organic Compounds †

Marius Rodner [1,*], Donatella Puglisi [1], Sebastian Ekeroth [2], Ulf Helmersson [2], Ivan Shtepliuk [3], Rositsa Yakimova [3], Andreas Skallberg [4], Kajsa Uvdal [4], Andreas Schütze [5] and Jens Eriksson [1]

[1] Applied Sensor Science Unit, IFM, Linköping University, 58183 Linköping, Sweden; donatella.puglisi@liu.se (D.P.); jens.eriksson@liu.se (J.E.)
[2] Plasma & Coatings Physics Division, IFM, Linköping University, 58183 Linköping, Sweden; sebastian.ekeroth@liu.se (S.E.); ulf.helmersson@liu.se (U.H.)
[3] Semiconductor Materials Division, IFM, Linköping University, 58183 Linköping, Sweden; ivan.shtepliuk@liu.se (I.S.); rositsa.yakimova@liu.se (R.Y.)
[4] Division of Molecular Surface Physics & Nanoscience, IFM, Linköping University, 58183 Linköping, Sweden; andreas.skallberg@liu.se (A.S.); kajsa.uvdal@liu.se (K.U.)
[5] Lab for Measurement Technology, Department of Systems Engineering, Saarland University, 66041 Saarbrücken, Germany; schuetze@lmt.uni-saarland.de
* Correspondence: marius.rodner@liu.se; Tel.: +46-700-896785

† This paper is an extended version of the conference paper: Iron Oxide Nanoparticle Decorated Graphene for Ultra-Sensitive Detection of Volatile Organic Compounds. In Proceedings of the EUROSENSORS 2018, Graz, Austria, 9–12 September 2018.

The operating temperature of 150 °C (~423 K) is high enough to provide efficient desorption of gas molecules during the measurements. To confirm this claim, we performed additional DFT frequency calculations for the following desorption reaction at 423 K (for example, benzene desorption):

$$C_6H_6@Fe_3O_4/EG \rightarrow C_6H_6+Fe_3O_4/EG$$

Such calculations allowed us to find the transition state for the reaction and, therefore, to estimate the activation energy (barrier) for benzene desorption. The transition state structure was confirmed by the presence of only one imaginary frequency at −68.38 cm^{-1}. The desorption barrier for benzene molecules on Fe_3O_4-decorated EG is only 0.167 eV, making benzene out-diffusion possible at 150 °C. Reaction energy profile for a benzene molecule is shown by Figure S1. Similar behavior is predicted for formaldehyde desorption.

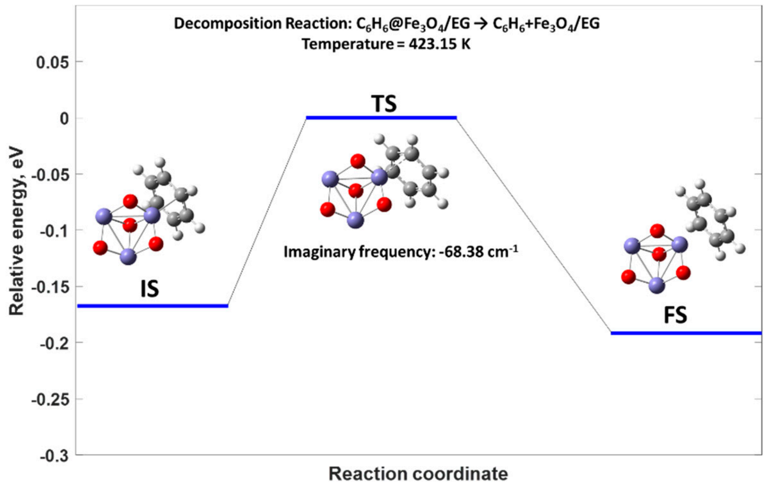

Figure 1. Reaction energy profile corresponding to benzene desorption. IS, TS and FS represent the initial state, transition state and final state, respectively.
